# CORESS feedback

**DOI:** 10.1308/003588413X13511609957975

**Published:** 2013-03

**Authors:** Frank CT Smith

## Abstract

Three of the cases in this issue of CORESS Feedback relate to failure of either giving or taking of information. A good clinical history underpins management decisions and emphasis on providing the general practitioner (and patient) with a comprehensive written discharge summary, describing treatment, is paramount. The final case illustrates once again that the role of the World Health Organization checklist and the ‘time-out’ cannot be overestimated in facilitating safe surgery.

We are grateful to the clinicians who have provided the material for these reports. The online reporting form is on our website (www.coress.org.uk), which also includes all previous Feedback Reports. Published contributions will be acknowledged by a ‘Certificate of Contribution’, which may be included in the contributor’s record of continuing professional development.

## Absent appendix (Ref 119)

A 32-year-old woman presented as an emergency with right iliac fossa pain and vomiting. She had a medical history of anorexia and bulimia as well as a laparoscopy for gynaecological reasons at another institution several months previously. On the morning after admission she was still tender with rebound pain on coughing. She gave no history of gynaecological or urinary problems. Ultrasonography undertaken six days previously had been normal. Computed tomography was undertaken, which was reported as normal although assessment of bowel loops was difficult.

With persisting right iliac fossa pain, raised leucocyte count and elevated C-reactive protein, a laparoscopy was undertaken for probable appendicitis. No abnormality was revealed in the pelvis, small or large bowel. However, where the appendix had been, there was a row of staples across the base. Postoperatively, the patient denied all knowledge of the previous appendicectomy. This patient was unaware she had had an appendicectomy and a potentially unnecessary procedure was undertaken.

### Reporter’s comments

With the move away from open surgery (involving a grid iron or Lanz surgical incision scar) to the generic scars of a laparoscopy, patients should be informed (preferably in writing) of any procedures undertaken laparoscopically.

### CORESS comments

This problem is not unique to laparoscopic surgery. Patients may not remember or fully understand what procedure has been performed for a variety of reasons. It is good practice to give patients a copy of the discharge letter that explains what procedure was undertaken and why. In this case, a diagnostic laparoscopy was not unreasonable. It is likely that staples from an appendicectomy would have shown up on computed tomography.

## Failure to recognise alcohol withdrawal in bleeding patient (Ref 123)

A 52-year-old man, who had suffered a nasal deformity from trauma sustained many years previously, underwent a routine septorhinoplasty. He had developed deep vein thromboses in the past and regular warfarin therapy had been stopped six days prior to surgery. He was covered with low molecular weight heparin in the perioperative period. Surgery was uneventful.

On the night following surgery, the patient bled and underwent nasal packing. In the morning, he was stable and warfarin was restarted as he was at risk of deep vein thrombosis. On the second postoperative day, he became very agitated, pulled out his packs and bled profusely. He became very disruptive, attempting to discharge himself. Subsequently, he became even more distressed and increasingly difficult to manage, such that the on-call psychiatrist was called. The patient was sectioned and sedated.

However, though bleeding continued, surgical staff were not called to the ward. At some point the patient went to the toilet and collapsed with a cardiac arrest. The resuscitation team was called and cardiopulmonary resuscitation commenced. After three cycles of resuscitation, cardiac output was restored and he was transferred to the intensive care unit for support. Subsequently, computed tomography demonstrated acute hypoxic brain injury. A belated careful history obtained from the family revealed that the cause of the patient’s postoperative confusion was likely to have been due to acute alcohol withdrawal.

### Reporter’s comments

There was failure to escalate care in a patient with ongoing bleeding and staff failed to recognise an acutely ill patient on the verge of collapse.

### CORESS comments

In acute bleeding, care needs to be escalated quickly and appropriately. A ‘watch and wait’ approach is not the right option. Careful assessment is important. In this case, the risk of bleeding was greater than the risk of deep vein thrombosis and anticoagulation could have been corrected in conjunction with further surgical exploration. Had a comprehensive history been obtained at the preoperative assessment, risk of acute alcohol withdrawal might have been recognised earlier, allowing appropriate management. The patient’s disruptive condition may have distracted medical and nursing staff from the potentially more serious problem of continued bleeding.

Specialist advice obtained from the CORESS Advisory Board stated:

The law on treatment of patients who cannot consent for themselves, or who suffer from acute mental disorder and who refuse consent, or who are incompetent to give consent, is complex and differs in some respects between England, Wales, Scotland and Northern Ireland. Patients who are suffering from a mental health disorder, and who present a danger to themselves or others, may be detained under the relevant mental health legislation, assessed and treated for that mental disorder and for its physical consequences. However, advice should be taken on a case-by-case basis on whether the patient is suffering from a mental disorder as defined within the legislation and whether treatment can be provided on that basis.

Under different legislation, the Mental Capacity Act in England and Wales or the Adults with Incapacity Act in Scotland, it is possible for attorneys to be appointed to provide consent on behalf of patients who cannot consent for themselves. Lastly, under common law, patients who are incapable of providing consent can be treated if that treatment is in their best interests.

## Antiembolic stockings compound leg ischaemia trouble (Ref 127)

A 75-year-old woman underwent an emergency Hartmann’s procedure for complicated diverticular disease. Three days postoperatively, she complained of pain in her left leg and foot. On removal of her antiembolic stockings, she was found to have a critically ischaemic leg. The on-call vascular surgeon arranged for magnetic resonance angiography, which confirmed the presence of a superficial femoral artery occlusion. This was treated successfully by angioplasty but the patient required emergency calf fasciotomies for compartment syndrome. The forefoot remained ischaemic and required partial amputation. On further questioning, she gave a history of progressive debilitating short distance intermittent claudication for two years prior to admission for surgery. No vascular examination of the legs was documented in the admission notes.

### Reporter’s comments

Antithrombotic compression stockings should *not* be applied if there is a history of or if there are signs of peripheral vascular disease of the lower limbs. Peripheral pulses should be assessed before prescribing thromboembolic deterrent stockings. Other factors contributing to ischaemia in this case may have included perioperative hypotension, legs elevated in stirrups and leg oedema.

### CORESS comments

A comprehensive medical history would have revealed symptoms of peripheral arterial disease and appropriate examination should have been undertaken. Risks of compression stockings in patients with arterial disease are well documented and in a patient with a history of claudication, preoperative measurement of ankle–brachial pressure index would have been appropriate. A venous thromboembolism assessment should have been conducted and perioperative subcutaneous heparin could have been employed as an alternative antithrombotic precaution. In a patient wearing antiembolic stockings, the legs should be examined regularly in the postoperative period.

## Surgical marking unseen (Ref 128)

I was operating on a morning list with three primary inguinal hernias under local anaesthesia (one right and two left). All patients were seen preoperatively on the ward, consented and the proposed side of surgery was marked. The previous week I had inadvertently marked a patient close to the incision site so this time I deliberately marked the side of operation higher on the abdomen. On arriving in theatre, I led a team briefing with all the theatre and anaesthetic staff.

The first patient was brought into theatre for hernia repair under local anaesthesia. He was given a small dose of sedation but remained relaxed and orientated. We talked about his family and business while I administered the local anaesthetic. Once the regional block was complete, we draped the patient and paused for our World Health Organization (WHO) ‘time-out’ check. Being awake, the patient even contributed to this by confirming his name and date of birth. It was during this check that a theatre healthcare assistant asked us to stop what we were doing and pointed out that we were about to operate on the wrong side. The pen mark denoting the side of operation had been covered by the surgical drapes.

I immediately explained to the patient what had occurred and discussed the case with the anaesthetist. We decided to place the patient at the end of the operating list to allow time for the mistakenly administered local anaesthetic to wear off. His procedure was performed later that morning without incident.

### Reporter’s comments

Two circumstances led to this error. While deliberately high to avoid the surgical field, the mark I had made was not visible when I exposed the patient to administer local anaesthetic. Second, the patient was sedated before the time-out check and, while alert, was chemically disinhibited. The fact that he was conscious and did not object to local anaesthetic being injected into the wrong side lulled me into a false sense of security. My practice has changed so that patients are now marked in conspicuous sites on the side of surgery and they are no longer sedated before the final time-out check has occurred. The importance of the timeout check was really highlighted to me in this case but I believe it was the brief before the list and the fostering of an environment where everyone felt comfortable to speak up that really saved the day.

### CORESS comments

The WHO checklist is effective and has been designed to reduce the incidence of adverse events such as wrong side surgery. Its use is strongly advocated. As with any checklist, however, there is a danger of overfamiliarity and merely paying lip service to the checks rather than using them as an effective tool. The value of the time-out in enhancing theatre team communication is evident in this report. The surgical mark should be visible even when the patient is draped. Concerns over risks of tattooing from surgical marking combined with incisions are not well founded.

